# Plasma free cortisol and b-type natriuretic peptide in septic shock

**DOI:** 10.1186/cc9830

**Published:** 2011-03-11

**Authors:** D Sturgess, B Venkatesh

**Affiliations:** 1 The University of Queensland, Brisbane, Australia

## Introduction

Previous studies of patients with septic shock have independently demonstrated alterations in plasma concentrations of b-type natriuretic peptide (BNP) and plasma free cortisol (PFC). Previous data suggest that a reciprocal relationship might exist. However, the relationship between these hormones in patients with septic shock is unclear. We sought to compare paired measurement of both BNP and PFC in a preliminary study of septic shock patients.

## Methods

Twenty-one consecutive adult patients from a tertiary-level, multidisciplinary ICU underwent blood collection within 72 hours of developing septic shock.

## Results

Mean ± SD APACHE III score was 80.1 ± 23.8. Hospital mortality was 29%. Log PFC demonstrated positive correlation with log BNP (*r *= 0.55; *P *= 0.019). Log PFC also correlated with APACHE III (*r *= 0.67; *P *< 0.001) and norepinephrine dose (*r *= 0.55; *P *= 0.01). APACHE III (*P *= 0.001) and norepinephrine dose (*P *= 0.02) were independent predictors of PFC. A model incorporating both variables explained 68% of variation in PFC (*R*^2 ^= 0.682).

## Conclusions

This preliminary study of patients with septic shock demonstrates a modest positive correlation between PFC and BNP concentration. The APACHE III score and norepinephrine dose were independent predictors of PFC.

